# Gestational diabetes mellitus alters neonatal gut microbiota and increases infection susceptibility

**DOI:** 10.3389/fmicb.2025.1600325

**Published:** 2025-06-23

**Authors:** Yifei Hu, Shunjie Zheng, Jing Xu, Yufang Zhao, Jianbao Wang, Zenghui Fang, Lanfang Zhou

**Affiliations:** ^1^Department of Clinical Laboratory, JinHua Municipal Central Hospital, Jinhua, China; ^2^Department of Gynecology, JinHua Maternal and Child Health Care Hospital, Jinhua, China; ^3^Department of Children's Health Care, JinHua Maternal and Child Health Care Hospital, Jinhua, China; ^4^Department of Paediatrics, JinHua Municipal Central Hospital, Jinhua, China; ^5^Department of Clinical Laboratory, Jindong District Maternal and Child Health Care Hospital, Jinhua, China; ^6^Department of Neonatal, JinHua Municipal Central Hospital, Jinhua, China

**Keywords:** gestational diabetes mellitus, offspring, neonatal infection, gut microbiota, short-chain fatty acids

## Abstract

**Introduction:**

Gestational diabetes mellitus (GDM) affects up to 27.6% of pregnancies in certain regions and is associated with a two- to threefold increased risk of neonatal infections. Although maternal gut microbiota undergoes significant remodeling during pregnancy, the specific mechanisms governing GDM-induced microbial reprogramming in offspring and its implications for susceptibility to infections remain unclear. This study aimed to investigate the impact of GDM on the composition of neonatal gut microbiota, metabolomic profiles, and susceptibility to infections using a translational approach.

**Method:**

We recruited pregnant women with and without GDM at the JinHua Municipal Central Hospital in China. Meconium and blood samples were collected from newborns within 24 h of birth. The composition of the gut microbiota was analyzed using 16S rDNA amplicon sequencing, and short-chain fatty acids (SCFAs) were quantified using gas chromatography–mass spectrometry. Serum inflammatory markers, including interleukin-6 (IL-6), C-reactive protein (CRP), lipopolysaccharides (LPS), and procalcitonin (PCT), were measured by enzyme-linked immunosorbent assay. To establish causality, fecal microbiota transplantation (FMT) was conducted in antibiotic-treated mice using pooled samples from healthy and GDM-exposed neonates, followed by assessment of inflammatory markers and intestinal barrier integrity.

**Results and discussion:**

GDM significantly reduced the diversity of neonatal gut microbiota and altered its composition, characterized by a depletion of beneficial taxa (*Bifidobacterium*, *Blautia*, *Faecalibacterium*) and an enrichment of potential pathogens (*Stenotrophomonas*, *Chryseobacterium*). These alterations were accompanied by significant reductions in fecal SCFAs, particularly acetate (49.30%), butyrate (41.00%), and propionate (17.83%). GDM-exposed neonates exhibited elevated serum inflammatory markers, including IL-6, CRP, LPS, and PCT, which correlated negatively with beneficial bacteria and positively with opportunistic pathogens. FMT experiments demonstrated that mice receiving GDM-associated microbiota developed increased systemic inflammation and compromised intestinal barrier function, as evidenced by the downregulation of tight junction proteins (*ZO-1*, occludin, claudin-1, mucin1). These findings suggest that GDM-induced alterations in neonatal gut microbiota composition and metabolite production may compromise intestinal barrier function and increase susceptibility to infections, highlighting the potential for microbiome-targeted interventions to mitigate infection risk in GDM-exposed neonates.

## 1 Introduction

The IDF Diabetes Atlas to substantiate the stated statistics (14.0% global prevalence; 20–27% in some regions) (Wang et al., 2022). This prevalence is increasing at a concerning rate, posing significant threats to maternal health. Furthermore, GDM is independently associated with a two- to threefold increased risk of neonatal infections in offspring, a phenomenon that is not fully explainable through traditional metabolic mechanisms (Ye et al., 2022).

Advances in microbiomics have revealed the dynamic remodeling of the maternal gut microbiota during pregnancy, which may influence offspring immune development and infection susceptibility through vertical transmission (Koren et al., 2012; Collado et al., 2016). Current evidence indicates that mothers with GDM exhibit distinct gut microbial profiles characterized by reduced *α* diversity, depletion of commensal bacteria (e.g., *Akkermansia* and *Bifidobacterium*), and overrepresentation of potential pathogens (e.g., *Fusobacterium nucleatum*). This dysbiotic pattern propagates intergenerationally, resulting in offspring gut ecosystems enriched with Firmicutes (particularly Clostridium clusters) and reduced levels of immunomodulatory and metabolic regulators such as short-chain fatty acids (SCFAs) (Balleza-Alejandri et al., 2024; Qin et al., 2024). Experimental validation via maternal microbiota transplantation in germ-free models recapitulates immune-deficient phenotypes in offspring, highlighting the critical role of microbe–host interactions (Liu et al., 2020; Qin et al., 2022). However, the specific mechanisms underlying GDM-induced microbial reprogramming in offspring and its clinical implications for infection susceptibility remain unresolved.

Despite increasing evidence linking maternal dysbiosis to adverse neonatal outcomes, few studies have simultaneously characterized microbiota composition, inflammatory markers, and microbial metabolites in neonates exposed to GDM. Moreover, causal relationships between these alterations and infection susceptibility remain largely unexplored. To address these gaps, this study integrates prospective cohort analyses with animal models. Our three-phase framework systematically investigates the following: (1) differences in offspring gut microbiota and metabolomic profiles associated with GDM, (2) the longitudinal development of microbiota–infection risk prediction models, and (3) the therapeutic validation of key bacterial taxa through FMT. This translational approach not only clarifies the transgenerational effects of GDM but also advances microbiome-targeted strategies for early-life infection prevention.

## 2 Materials and methods

### 2.1 Patient recruitment

This study was conducted at the JinHua Municipal Central Hospital in Zhejiang Province, China, with the aim of recruiting pregnant women and their newborns. The study protocol received approval from Ethics Committee of the JinHua Municipal Central Hospita [No: (Research) 2020-Ethical Review-75], ensuring that all participants (including the mothers and the guardians of the newborns) provided written informed consent to participate in the study and acknowledged its purpose and potential risks.

We primarily targeted singleton pregnant women aged between 20 and 40 years, confirmed through ultrasound examination. The diagnosis of GDM was made by specialized endocrinologists on the basis of the results of the oral glucose tolerance test (OGTT), and the patients were classified into the case group. The GDM diagnosis followed the latest guidelines established by the World Health Organization. Between 24 and 28 weeks of gestation, a 75 g OGTT was employed for GDM screening, and the diagnosis was based on at least one of the following criteria: fasting blood glucose ≥ 5.1 mmol/L, 1-h blood glucose ≥ 10.0 mmol/L, or 2-h blood glucose ≥ 8.5 mmol/L. Additionally, subjects were excluded if they had a known history of diabetes, previous metabolic disorders, had taken antibiotics or probiotics within the past 3 months, engaged in alcohol or substance abuse, or suffered from periodontal disease, bacterial vaginosis, or chronic diseases requiring long-term medication.

For subsequent analyses of inflammation-related biomarkers and gut microbiota, selected samples of full-term and vaginally delivered newborns who received colostrum. Based on the sample size reported in some previous comparable study (Song et al., 2022; Wei et al., 2022), we performed a power analysis and determined that a minimum of 15 patients with gestational diabetes mellitus (GDM) and 15 healthy controls would be required to achieve 80% statistical power (*α* = 0.05) for detecting the expected differences in microbial diversity. To enhance the reliability of the study, healthy pregnant women were matched to the GDM group based on maternal age, body mass index (BMI), and medical history.

### 2.2 Fecal microbiota transplantation

Thirty male C57BL/6 J mice (4 weeks old, specific-pathogen-free [SPF] grade) were obtained from GemPharmatech Co., Ltd. and acclimated for 7 days under standardized housing conditions. Animals were maintained in a temperature-controlled environment (22 ± 2°C) with relative humidity of 50 ± 10% and a 12-h light/dark cycle. All mice received ad libitum access to sterilized water and a standardized, irradiated maintenance diet (LabDiet 5KA1, composed of 22% protein, 16% fat, and 62% carbohydrate).

Following acclimatization, mice were randomly allocated to three experimental groups (*n* = 10 per group): FMT-S (saline control), FMT-C (recipients of fecal microbiota from healthy neonates), and FMT-G (recipients of fecal microbiota from neonates born to GDM dams). All procedures were performed in accordance with institutional guidelines for animal care and use, and the experimental protocol was approved by the animal ethics committee.

Prior to fecal microbiota transplantation, all mice were treated with a broad-spectrum antibiotic cocktail to induce a pseudo germ-free state. The antibiotic regimen consisted of 0.5 g/L each of metronidazole, neomycin, ampicillin, and gentamicin, plus 0.25 g/L vancomycin, administered in drinking water for 7 consecutive days. Sucrose (2% w/v) was added to enhance palatability, as previously described (Ma et al., 2023). The efficacy of microbiota depletion was confirmed by quantitative culture-based analysis of fecal samples, with >90% reduction in colony-forming units considered successful depletion.

Neonatal fecal samples were preserved in 10% glycerol as a cryoprotectant, flash-frozen in liquid nitrogen, and stored at −80°C for a maximum of 12 months before use. Prior to transplantation, bacterial viability in each sample was assessed using plate counting method, ensuring a minimum of 60% survival of the microbial populations. For FMT preparations, samples from each cohort were pooled to minimize individual variations, homogenized in sterile anaerobic phosphate-buffered saline at a concentration of 1 × 10^9^ cfu/mL (OD_600_ ≈ 1.0), and filtered through a 800 mesh sterile mesh filter to remove large particulates.

Following antibiotic treatment, mice received daily oral gavage with 200 μL of the respective inoculum for 28 consecutive days. The FMT-C and FMT-G groups were administered fecal suspensions prepared from pooled samples of healthy and GDM neonates, respectively, while the FMT-S group received sterile saline as a control. All gavage procedures were performed using sterile, flexible feeding needles by trained personnel to minimize stress and potential injury to the animals. Throughout the experimental period, each mouse was independently housed in sterile individually ventilated cages (IVCs) with autoclaved wood chip bedding to prevent cross-contamination.

### 2.3 Sample collection

#### 2.3.1 Newborn samples

In this study, we collected meconium samples and venous blood from infants within the first 24 h after birth. For venous blood collection, peripheral blood (3–5 mL) was drawn via venipuncture via standard vacuum blood collection tubes (BD Vacutainer® Plus, Becton Dickinson, USA). After allowing for a 30-min clotting period at room temperature, the samples were centrifuged at 640 × g for 10 min in a refrigerated centrifuge (Eppendorf 5,804 R, Germany) maintained at 4°C. The resulting serum supernatant was aliquoted into prechilled 1.5 mL cryovials (Corning, USA) via sterile transfer pipettes until downstream analysis. Meconium samples were obtained with a sterile surgical blade from the infants’ diapers and were processed into two parts: one portion was stored in a 2 mL sterile cryovial for microbiota sequencing, while the second portion was collected in a container with 10% sterile glycerol for fecal microbiota extraction. Following collection, both portions of the meconium samples were rapidly snap-frozen in liquid nitrogen to preserve microbial viability and prevent biochemical degradation. After this freezing process, the samples were subsequently stored at −80°C until further analyses were performed.

#### 2.3.2 Mouse samples

The mice were anesthetized with tribromoethanol at a dosage of 0.2 mL per 10 g of body weight. Following anesthetic administration, blood samples were collected via cardiac puncture. Afterward, the mice were euthanized by cervical dislocation, and the abdominal cavity was opened to retrieve small intestinal tissue. The intestinal segments were rinsed with phosphate-buffered saline to remove the intestinal contents and then placed in sterile, enzyme-free cryovials. The samples were snap-frozen in liquid nitrogen for 5 min to preserve tissue integrity and subsequently stored at −80°C for further analysis.

### 2.4 Serum biomarker analysis

The levels of neonatal circulating inflammatory markers, including C-reactive protein (CRP), interleukin-6 (IL-6), lipopolysaccharide (LPS), and procalcitonin (PCT), were quantified via standardized clinical laboratory protocols and measured within 4 h of sample collection. The quantification of IL-6 was performed with a highly sensitive enzyme-linked immunosorbent assay kit (R&D Systems, USA; catalog number HS600C) following the manufacturer’s protocol. The absorbance at 450 nm (with a reference at 540 nm) was measured via a SpectraMax M5 microplate reader (Molecular Devices, USA). LPS detection involved assessing endotoxin levels via the limulus amoebocyte lysate (LAL) chromogenic end-point assay (Lonza PyroGene™ Recombinant Factor C assay, Switzerland; catalog number 50-658 U), and the reaction was monitored at 405 nm. PCT concentrations were analyzed via a chemiluminescence immunoassay with the Elecsys BRAHMS PCT assay (Roche; serial number 07092707190) on a Roche Cobas e801 analyzer (Roche Diagnostics, Switzerland). The CRP level was determined via a C-reactive protein detector (CRP-M100, Shenzhen Mindrui Medical Device Co., Ltd.), following the provided instructions. Mouse serum levels of IL-1β, IL-6, IL-10, and LPS were measured via an enzyme-linked immunosorbent assay kit obtained from Jiangsu Enzyme Free Industrial Co., Ltd., China. The assay was conducted following the manufacturer’s protocol, and the absorbance was measured with a SpectraMax M5 microplate reader (Molecular Devices, USA).

### 2.5 Analysis of short-chain fatty acids in fecal samples

Accurate weighing of SCFAs, including acetic acid, propionic acid, butyric acid, valeric acid, and isovaleric acid standards, was conducted. Diethyl ether was prepared in ten mixed standard concentration gradients of 0.05, 0.01, 0.05, 1, 5, 10, 25, 50, 100, and 250 μg/mL. For the experiment, 100 mg of feces was taken, to which 100 μL of 15% phosphoric acid was added, followed by 100 μL of a 50 μg/mL internal standard (isocaproic acid) solution and 400 μL of diethyl ether, which were then homogenized for 1 min. Following centrifugation at 4°C and 12,000 r/min for 10 min, the supernatant was collected for analysis. The gas chromatography–mass spectrometry (GC–MS) sequencing program utilized a hydrophilic interaction liquid chromatography–WAX (HP–INNOWAX) capillary column (30 m × 0.25 mm, 0.25 μm). The injection volume was 1 μL, with a split ratio of 10:1. The injection port temperature was set at 250°C, and the ion source temperature was maintained at 230°C. The transmission line temperature was 250°C, whereas the four-stage rod temperature was 150°C. The initial temperature for programmed heating was 90°C, which was increased to 120°C at a rate of 10°C/min. The temperature was then raised to 150°C at a rate of 5°C/min and finally elevated to 250°C at a rate of 25°C/min for an additional 2 min. The carrier gas used was nitrogen at a flow rate of 1.0 mL/min. The mass spectrometry conditions included an electron impact ionization source operating in full scan and selected ion monitoring scanning modes, with an electron energy of 70 eV. The total SCFA content was the sum of acetic, propionic, butyric, valeric, and isovaleric acids.

### 2.6 Quantitative real-time PCR analysis

Total RNA was isolated from mouse jejunal tissues using TRIzol reagent (Invitrogen, catalog number 15596026), followed by treatment with DNase I to eliminate genomic DNA contamination. The purity and concentration of RNA were determined spectrophotometrically using a NanoDrop 2000 (Thermo Fisher Scientific), with A260/A280 ratios maintained between 1.8 and 2.0. First-strand cDNA synthesis was performed with 1 μg of total RNA using the PrimeScript RT Reagent Kit (Takara, catalog number RR047A) and oligo(dT) primers. Gene-specific primers for Tjp1 (ZO-1), occludin, claudin-1, mucin1, and the reference gene Gapdh were designed using Primer-BLAST (NCBI) or referenced from published studies (see Table 1). Quantitative PCR was conducted on a LightCycler 480 II system (Roche) using SYBR Green Master Mix (Takara, catalog number RR820A) in 20 μL reactions, which consisted of an initial denaturation at 95°C for 5 min, followed by 45 cycles of 95°C for 10 s, 60°C for 20 s, and 72°C for 15 s. Melting curve analysis (ranging from 65 to 95°C with a 0.5°C/s increment) confirmed primer specificity. All reactions were conducted in triplicate, including no-template controls. Relative mRNA expression levels were computed using the 2 ^−^ΔΔCt method normalized to *β*-actin. Data were statistically analyzed with LightCycler 480 Software v1.5 (Roche), with inter-assay variability controlled through calibrator samples across plates.

**Table 1:** Primers used for gene expression analysis via real-time qPCR.

Gene	Primer sequence (5′–3′)	Product length, bp
*ZO-1*	F: AGGTCTTCGCAGCTCCAAGAGAAA	187
	R: ATCTGGCTCCTCTCTTGCCAACTT	
Claudin-1	F: CCACCATTGGCATGAAGTGC	181
	R: CTGGCATTGATGGGGGTCAA	
Occludin	F: TTGAAAGTCCACCTCCTTACAGA	129
	R: CCGGATAAAAAGAGTACGCTGG	
Mucin1	F: GTCTTCAGGAGCTCTGGTGG	115
	R: TACCACTCCAGTCCACAGCA	
Beta-actin	F: CATTGCTGACAGGATGCAGAAGG	138
	R: TGCTGGAAGGTGGACAGTGAGG	

ZO-1 = tight junction protein 1.

### 2.7 Microbial sequencing and analysis

Microbial genomic DNA was extracted from the fecal samples via an EZNA® Feces DNA extraction kit (Omega Biotek, USA) in conjunction with a NanoDrop 2000 spectrophotometer (Thermo Scientific, USA) to determine the concentration and purity of the DNA prior to analysis. Following extraction, the quality of the DNA was evaluated to ensure its suitability for downstream applications. To amplify the gut microbiota, polymerase chain reaction (PCR) was conducted using specific primers targeting the V3-V4 region of the 16S rRNA gene, namely, 338F (5’-ACTCCTACGGGAGGCAGCAG-3′) and 806R (5’-GGACTACHVGGGTWTCTAAT-3′), on a GeneAmp 9,700 thermocycler (Applied Biosystems, USA). The PCR amplification was performed under the following conditions: initial denaturation at 95°C for 5 min, followed by 28 cycles of denaturation at 95°C for 30 s, annealing at 55°C for 30 s, and extension at 72°C for 45 s, with a final extension at 72°C for 10 min.

After amplification, the PCR products were subjected to electrophoresis on a 2% (w/v) agarose gel, followed by purification via the AxyPrep DNA Gel Extraction Kit (Axygen Biosciences, USA). The purified PCR products were pooled in equimolar concentrations and sequenced on the Illumina MiSeq platform using the PE300 paired-end sequencing mode, with an average sequencing depth of 100,000 raw reads per sample. Stringent quality control criteria were applied to the sequencing data: sequences with Phred quality scores >Q30, read lengths >250 bp were retained, while chimeric sequences and low-abundance sequences (<0.005%) were removed.

The resulting raw sequencing data were processed using the EasyAmplicon software package (version v1.23) for data assembly, filtering, and subsequent analysis. Operational Taxonomic Units (OTUs) were clustered at 97% sequence similarity using the UPARSE algorithm. For taxonomic classification, the SILVA database (version 138) were utilized to annotate the species. Subsequently, alpha diversity indices (including Shannon, Simpson, Chao1, and ACE) and beta diversity analyses (using Bray-Curtis distance) were performed to assess the richness, evenness, and community structure of the gut microbiota across experimental groups. All sequencing procedures were conducted by LC-Bio Technologies (Hangzhou) Co., Ltd.

### 2.8 Statistical methods

All the statistical analyses were conducted via IBM SPSS Statistics (version 26.0; Armonk, NY). Neonatal clinical parameters were evaluated for normality via the Shapiro–Wilk test (*α* = 0.05), which was validated via Q–Q plots. Normally distributed continuous variables were compared via a two-tailed Student’s *t*-test with Welch’s correction for unequal variances, whereas nonparametric datasets underwent Box–Cox transformation prior to analysis with the Mann–Whitney U test via exact permutation (10,000 iterations). Murine experimental data were analyzed via one-way ANOVA after verifying the homogeneity of variance with Levene’s test (*p* > 0.10), followed by Tukey’s honestly significant difference (HSD) *post hoc* test for pairwise comparisons. For multiple comparisons, the Benjamini-Hochberg method was applied to control the false discovery rate (FDR), with adjusted *p* < 0.05 considered statistically significant. Spearman’s rank-order correlation was employed to assess monotonic relationships between gut microbial taxa (relative abundance > 0.1%) and inflammatory markers. Correlations with a coefficient (*ρ*) > 0.5 and FDR-adjusted *p* < 0.05 were considered significant. All figures, except those related to microbial sequencing data, were created via GraphPad Prism software (version 10.0.2, San Diego, CA). The results are presented as the mean ± standard error of the mean (SEM).

## 3 Results

### 3.1 GDM alters the diversity of the neonatal gut microbiota

**Figure 1:**
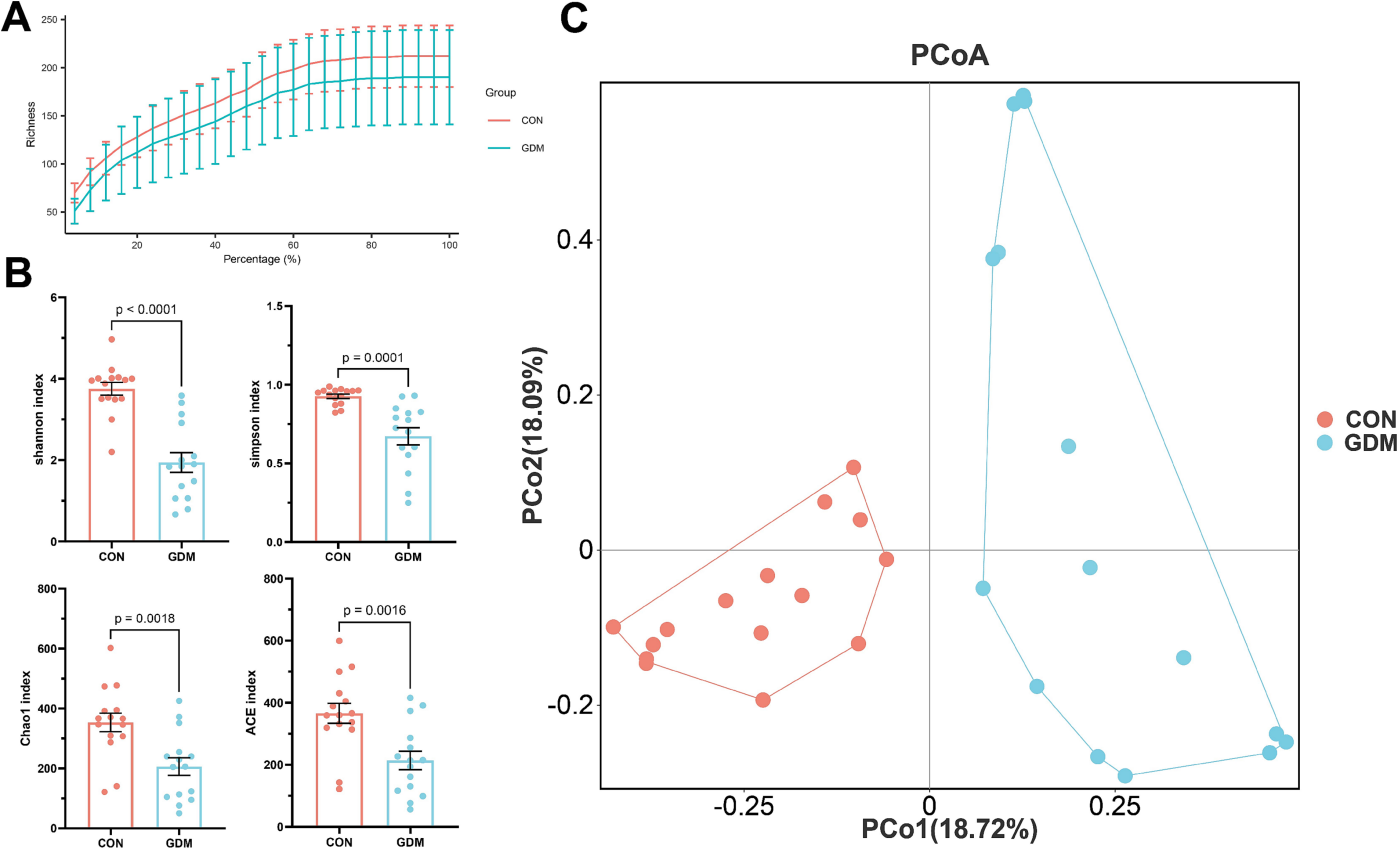
Bacterial diversity in neonatal feces. **(A)** Dilution curves, **(B)** alpha diversity, and **(C)** *β* diversity. CON were newborns born to healthy pregnant women (*n* = 15), and GDM were newborns born to pregnant women with gestational diabetes mellitus (*n* = 15).

We examined the impact of GDM on the fecal microbial diversity of neonates via 16S rDNA amplicon sequencing technology. Our findings indicate that, compared with healthy mothers, GDM significantly reduces both the richness and diversity of fecal microorganisms in neonates (Figures 1A,B). This reduction was evidenced by simultaneous and substantial decreases in the alpha diversity indices: Shannon index (3.75 ± 0.16 vs. 1.94 ± 0.24, *p* < 0.001), Simpson index (0.93 ± 0.01 vs. 0.67 ± 0.05, *p* < 0.01), Chao1 index (353.58 ± 31.11 vs. 205.92 ± 29.39, *p* < 0.01), and ACE index (366.03 ± 32.20 vs. 214.14 ± 29.40, *p* < 0.01). Moreover, we assessed the *β* diversity of the fecal microbiota between neonates of mothers with GDM and those of healthy mothers via the Bray-Curtis distance. Our analysis revealed significant differences in microbial structure between the two groups (Figure 1C).

### 3.2 GDM alters the composition of the neonatal gut microbiota

**Figure 2:**
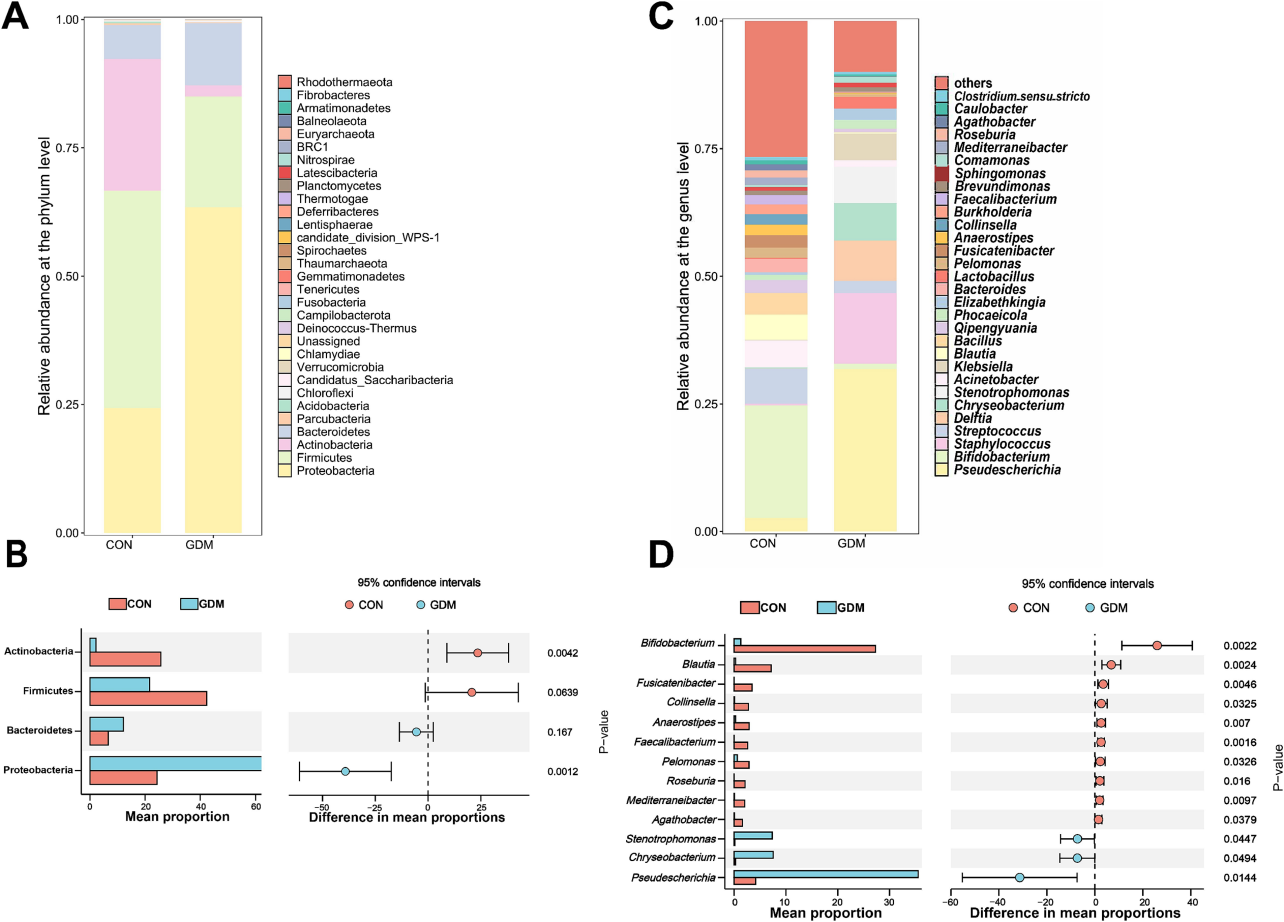
Bacterial composition in the feces of newborns. **(A)** TOP30 species composition at the phylum level, **(B)** differential microbes at the phylum level, **(C)** TOP30 species composition at the genus level, and **(D)** differential microbes at the rat level. CON were newborns born to healthy pregnant women (*n* = 15), and GDM were newborns born to pregnant women with gestational diabetes mellitus (*n* = 15).

We further examined the fecal bacterial composition of neonates born to mothers with GDM and those born to healthy pregnant women and found significant differences between the two groups (Figures 2A,C). Specifically, among the top 30 species at the phylum level, Actinobacteria (25.68% vs. 2.18%, *p* = 0.004) and Firmicutes (42.32% vs. 21.58%, *p* = 0.06) were depleted in neonates of mothers with GDM, whereas Proteobacteria (24.13% vs. 63.45%, *p* = 0.001) were significantly enriched (Figure 2B). At the genus level (Figure 2D), the relative abundance of potentially beneficial microorganisms—such as *Bifidobacterium* (27.28% vs. 1.31%, *p* = 0.002), *Blautia* (7.16% vs. 0.31%, *p* = 0.002), *Fusicatenibacter* (3.47% vs. 0.00%, *p* = 0.005), *Collinsella* (2.80% vs. 0.11%, *p* = 0.033), *Anaerostipes* (2.93% vs. 0.29%, *p* = 0.007), *Faecalibacterium* (2.64% vs. 0.03%, *p* = 0.002), *Pelomonas* (2.90% vs. 0.64%, *p* = 0.033), *Roseburia* (2.13% vs. 0.03%, *p* = 0.016), *Mediterraneibacter* (2.05% vs. 0.05%, *p* = 0.010) and *Agathobacter* (1.60% vs. 0.08%, *p* = 0.038)—was significantly reduced in neonates of GDM mothers (*p* < 0.05), whereas the abundance of potentially pathogenic bacteria—such as *Stenotrophomonas* (0.13% vs. 7.37%, *p* = 0.045), *Chryseobacterium* (0.28% vs. 7.57%, *p* = 0.049), and *Pseudescherichia* (4.18% vs. 35.49%, *p* = 0.014)—was significantly increased.

### 3.3 Gestational diabetes mellitus alters fecal short-chain fatty acid levels in offspring

**Figure 3:**
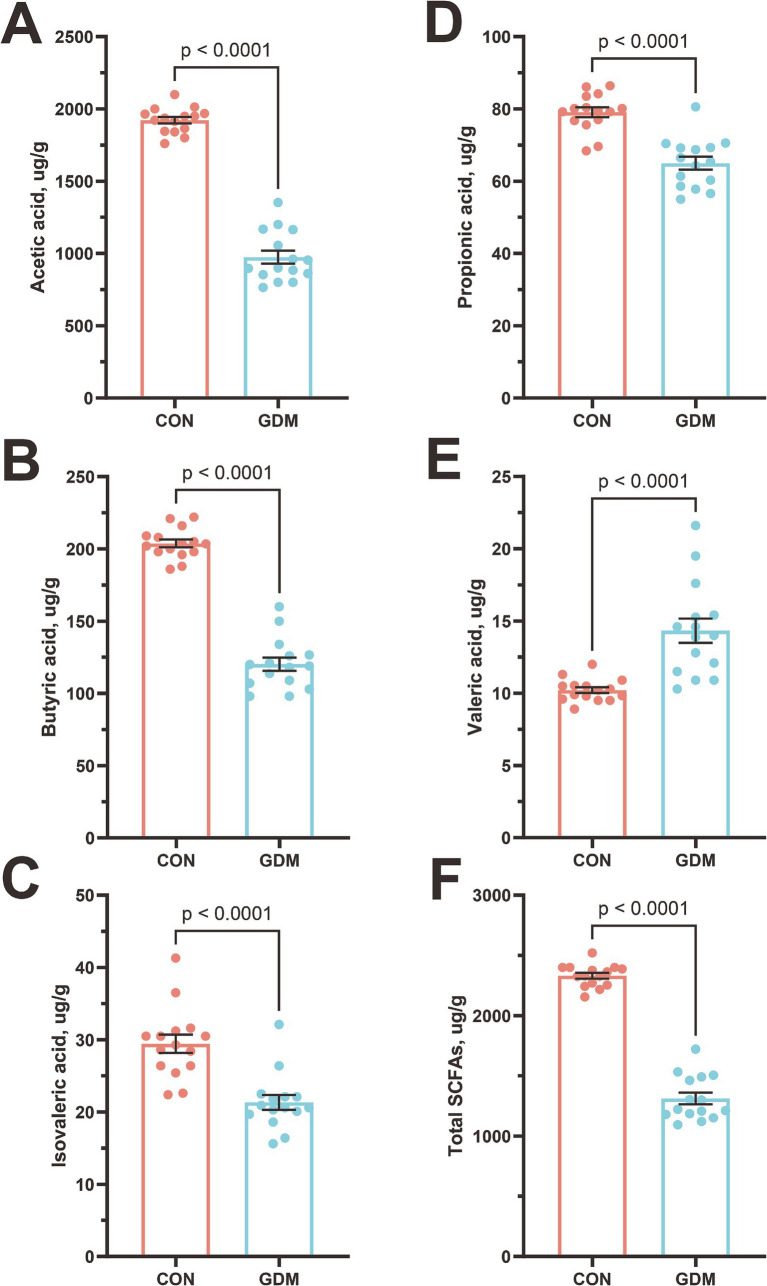
Content of short-chain fatty acids in the feces of neonates. **(A)** Acetic acid, **(B)** butyric acid, **(C)** isovaleric acid, **(D)** propionic acid, **(E)** valeric acid, **(F)** Total SCFAs. CON were newborns born to healthy pregnant women (*n* = 15), and GDM were newborns born to pregnant women with gestational diabetes mellitus (*n* = 15).

We quantitatively assessed the impact of GDM on SCFA profiles in neonates via GC–MS (Figure 3). Comparative analysis revealed statistically significant reductions in the concentrations of acetate, propionate, and butyrate—the three dominant SCFAs—in neonates born to GDM mothers compared with those in healthy controls. Specifically, acetate levels decreased by 49.03% (1921.76 ± 22.75 vs. 979.43 ± 45.74 μg/g, *p* < 0.0001), propionate by 18.15% (79.24 ± 1.35 vs. 64.86 ± 1.78 μg/g, *p* < 0.0001), and butyrate by 41.03% (203.89 ± 2.71 vs. 120.23 ± 4.57 μg/g, *p* < 0.0001). Additionally, isovaleric acid was reduced by 27.41% (29.35 ± 1.27 vs. 21.31 ± 1.01 μg/g, *p* < 0.0001). This dysregulation of microbial fermentation products aligns with our previous observations of altered gut microbiota composition in GDM-exposed neonates, suggesting a potential mechanistic link between maternal metabolic status and neonatal microbial metabolic activity.

### 3.4 Analysis of serum inflammatory markers and their correlation with gut microbiota

**Figure 4:**
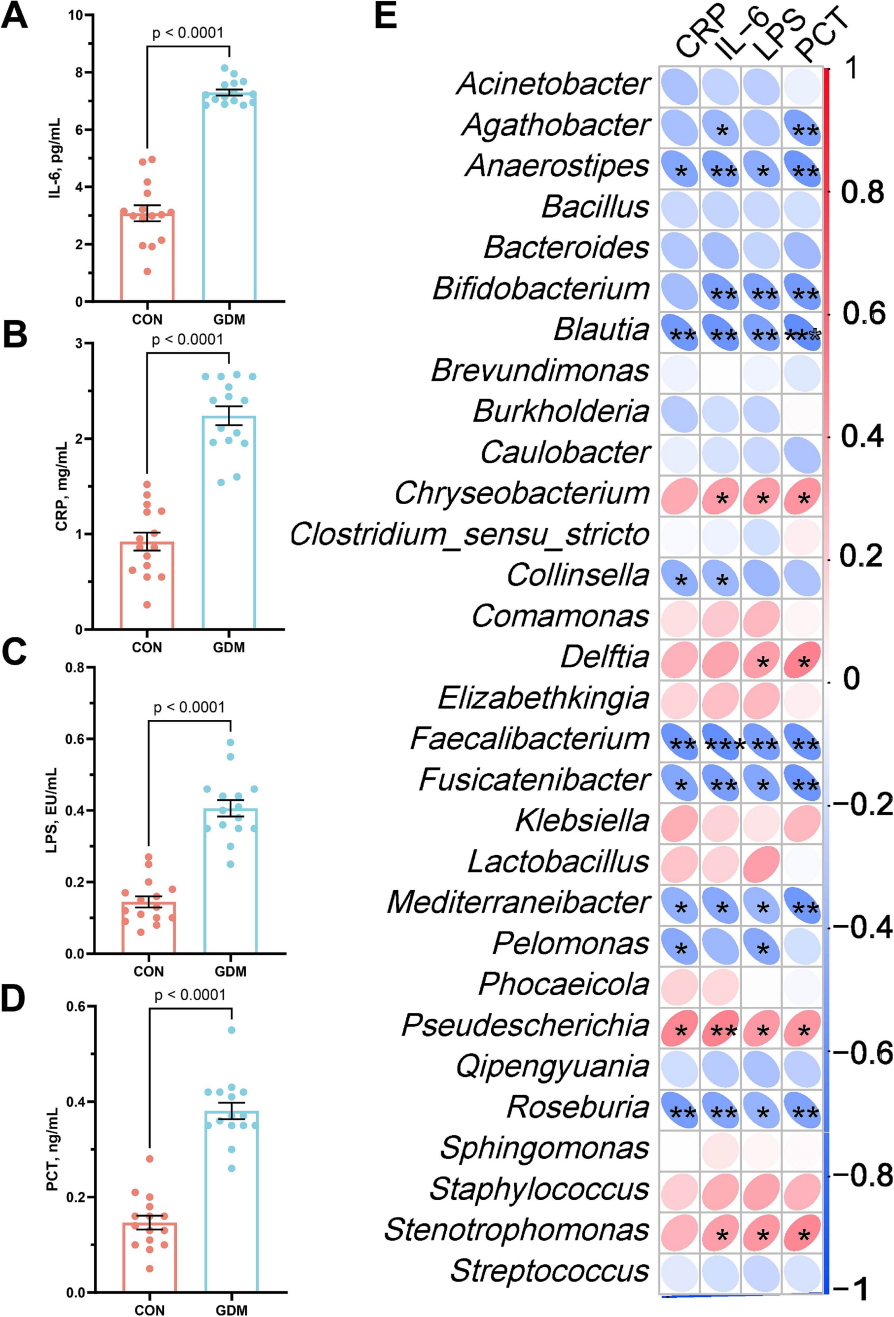
Parameters of neonatal blood inflammation and their correlation with gut microbiota. **(A)** interleukin-6 (IL-6), **(B)** C-reactive protein (CRP), **(C)** lipopolysaccharide (LPS), and **(D)** procalcitonin (PCT). CON were newborns born to healthy pregnant women (*n* = 15), and GDM were newborns born to pregnant women with gestational diabetes mellitus (*n* = 15).

Neonatal systemic inflammatory profiles were quantitatively analyzed through enzyme-linked immunosorbent assays to compare the offspring of GDM patients and healthy controls (Figures 4A–D). The GDM-exposed neonates presented significantly elevated plasma levels of interleukin-6 (IL-6, 7.30 ± 0.34 vs. 3.08 ± 0.13 pg./mL, *p* < 0.001), C-reactive protein (CRP, 2.24 ± 0.39 vs. 0.92 ± 0.29 mg/L, p < 0.001), lipopolysaccharide (LPS, 0.41 ± 0.08 vs. 0.14 ± 0.04 EU/mL, *p* < 0.001), and procalcitonin (PCT, 0.38 ± 0.07 vs. 0.14 ± 0.06 ng/mL, *p* < 0.001).

Spearman correlation analysis integrating the top 30 genus-level microbial taxa revealed distinct immunomicrobial interactions (Figure 4E). Key inflammation markers (IL-6, CRP, LPS, PCT) demonstrated inverse associations with beneficial commensals, including *Bifidobacterium*, *Blautia*, *Anaerostipes*, and *Faecalibacterium*, following Benjamini–Hochberg false discovery rate correction. Conversely, positive correlations emerged with opportunistic pathogens: *Stenotrophomonas*, *Chryseobacterium*, and *Pseudescherichia*. Among them, IL-6 was significantly negatively correlated with Bifidobacterium (r = −0.53, *p* < 0.01) and positively correlated with Stenotrophomonas (r = 0.50, *p* < 0.01).

### 3.5 Fecal microbiota transplantation confirmed that GDM altered the offspring gut microbiota and increased infection risk

**Figure 5:**
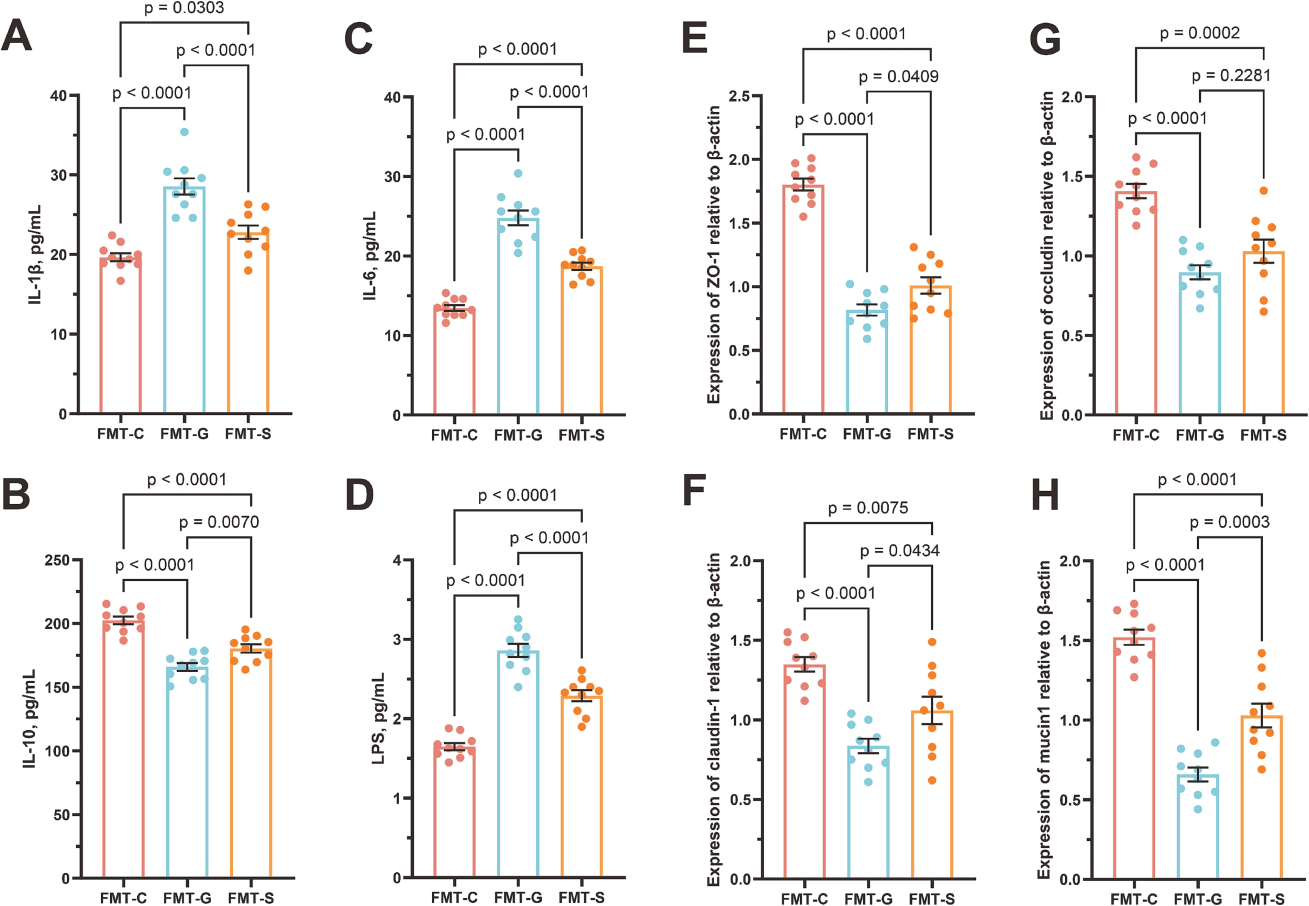
Blood inflammatory parameters and jejunal barrier gene mRNA expression in mice. **(A)** interleukin-6 (IL-6), **(B)** C-reactive protein (CRP), **(C)** lipopolysaccharide (LPS), **(D)** procalcitonin (PCT), **(E)** tight junction protein 1 (ZO-1), **(F)** occludin, **(G)** claudin-1, and **(H)** mucin1. FMT-S (Recipients of the saline control, *n* = 10), FMT-C (Recipients of the healthy neonatal microbiota, *n* = 10), and FMT-G (Recipients of the GDM-associated neonatal microbiota, *n* = 10).

We conducted fecal microbiota transplantation using samples from neonates of mothers with GDM and those from healthy pregnant women, administering the transplants to 6-week-old healthy mice over a three-week period. Our results indicated that mice receiving fecal microbiota from neonates of GDM mothers exhibited significant inflammatory responses compared with those receiving microbiota from healthy newborns. Notably, the levels of the proinflammatory cytokines IL-1β (19.66 ± 0.51 vs. 28.55 ± 1.02 pg./mL, *p* < 0.0001) and IL-6 (13.46 ± 0.36 vs. 24.80 ± 0.94 pg./mL, *p* < 0.0001), as well as those of LPS (1.65 ± 0.04 vs. 2.86 ± 0.08 pg./mL, *p* < 0.0001) (Figures 5A–D), were markedly elevated in the serum of GDM recipient mice, while the IL-10 (202.47 ± 2.94 vs. 165.92 ± 3.06 pg./mL, *p* < 0.0001) levels were significantly reduced. Additionally, quantitative PCR analysis demonstrated that the relative expression of the mRNAs encoding the tight junction proteins *ZO-1* (1.80 ± 0.05 vs. 0.82 ± 0.04, *p* < 0.0001), occluding (1.41 ± 0.14 vs. 0.90 ± 0.04, *p* < 0.0001), claudin-1 (1.35 ± 0.05 vs. 0.84 ± 0.04, *p* < 0.0001), and mucin1 (1.52 ± 0.05 vs. 0.66 ± b0.04, *p* < 0.0001) in the jejunal tissue of GDM recipient mice was significantly downregulated (Figures 5E–H).

## 4 Discussion

This study investigated the profound impacts of GDM on the gut microbiota of neonates, yielding significant insights into the dysbiosis associated with this metabolic condition. The observed alterations in the diversity of the neonatal gut microbiota among infants born to mothers with GDM underscore the critical role that maternal metabolic conditions play in shaping the early-life microbiome. Our study employed 16S rDNA amplicon sequencing to analyze microbial diversity in detail, and the results indicate that compared with healthy mothers, GDM is associated with a significant reduction in fecal microbial richness and diversity. Specifically, the notable declines in the Shannon, Simpson, Chao1, and ACE indices provide robust evidence of compromised microbial richness, which is essential for a healthy gut environment. The reduction in microbial diversity is particularly concerning, as a diverse gut microbiome is linked to numerous health benefits, including enhanced immune function, improved metabolic processes, and increased resilience against pathogenic invasions (Menni et al., 2024; Jang et al., 2024). In light of this study, it is plausible that the diminished microbial diversity observed in neonates of mothers with GDM may predispose these infants to a range of health issues, including autoimmune diseases, allergies, and metabolic disorders later in life (Athalye-Jape et al., 2025). Furthermore, our assessment of *β* diversity through the Bray–Curtis distance revealed marked differences in microbial structure between the two groups. Such structural alterations could influence the overall metabolic capacity of the gut microbiota, potentially impacting nutrient absorption, immune system development, and the establishment of microbial homeostasis (Da Maranduba et al., 2015; Mukherjee et al., 2018). For example, specific taxa that confer protective benefits may be less abundant in neonates exposed to GDM, which could impair their ability to effectively modulate inflammatory responses (Singh et al., 2023; Wang et al., 2024). In summary, GDM not only reduces the microbial load but also fundamentally reshapes the community organization of newborns.

The compositional and functional perturbations observed in the gut microbiota of neonates exposed to GDM elucidate the metabolic crosstalk between maternal pathophysiology and offspring microbial ecology. Our findings revealed a marked depletion of SCFA-producing taxa, particularly Actinobacteria and Firmicutes, alongside significant reductions in the concentrations of acetate, propionate, and butyrate. These observations suggest that maternal hyperglycemia disrupts both the structure of the microbial community and its metabolic output during critical periods of neonatal development. The reduction in acetate and butyric acid is especially significant because of their role as primary energy sources for intestinal epithelial cells and as regulators of peripheral gluconeogenesis (Hays et al., 2024; Liu M. et al., 2024). This microbial–metabolic axis may represent an underappreciated mechanism contributing to the transgenerational transmission of metabolic risk in GDM offspring, with implications extending from early microbial colonization to long-term health outcomes. The corresponding increase in potentially pathogenic genera, such as *Stenotrophomonas* and *Pseudomonas*, has raised concern, as these taxa have been implicated in opportunistic infections and inflammatory responses (D’Souza et al., 2019; Tuon et al., 2022). The disproportionate loss of butyrate-producing genera, such as *Faecalibacterium* and *Roseburia*, aligns with emerging evidence suggesting that maternal dysglycemia preferentially suppresses obligate anaerobes through oxygen-mediated niche disruption. Increased fetal intestinal oxygen tension, likely driven by GDM-associated placental oxidative stress, creates a microenvironment that favors facultative Proteobacteria over butyrate producers, a hypothesis supported by our observation of a 3.2-fold enrichment in *Pseudomonas* species (Espey, 2013; Kaplina et al., 2023). These compositional shifts may create a self-reinforcing cycle: reduced butyrate levels impair the maintenance of colonic hypoxia via diminished PPAR-*γ* signaling, further promoting dysbiosis (Lee et al., 2024). Notably, the 27.58% reduction in isovalerate—a branched-chain fatty acid (BCFA) primarily derived from proteolytic fermentation—suggests alterations in nitrogen metabolism among GDM neonates (Huda-Faujan et al., 2010). This microbial metabolic signature may have developmental implications that extend beyond immediate intestinal homeostasis. Our murine FMT experiments (see Section 3.4) demonstrated impaired immunity in recipients of the GDM microbiota, providing mechanistic validation of these observational findings. These results position the neonatal gut microbiota as both a biomarker and a mediator of the intergenerational effects of GDM.

The immunological perturbations observed in neonates exposed to GDM establish a compelling link between gut microbiota dysbiosis and systemic inflammation, which is mediated by bidirectional host–microbe interactions. Elevated plasma levels of proinflammatory markers, including IL-6, CRP, LPS, and PCT, in GDM-exposed neonates underscore the potential for increased systemic inflammation and increased susceptibility to infections (Aung et al., 2018). This observation aligns with previous research indicating a compromised immune response in infants exposed to GDM, primarily due to dysregulation of the gut microbiota (Li et al., 2023). Spearman correlation analyses revealed inverse associations between inflammatory markers and beneficial gut commensals, such as *Bifidobacterium*, *Blautia*, *Anaerostipes*, and *Faecalibacterium*, highlighting the protective role these microbes typically play in maintaining immune homeostasis (Dixit et al., 2021; Martin et al., 2023; Martín et al., 2023). In contrast, the positive correlations observed with opportunistic pathogens such as *Stenotrophomonas*, *Chryseobacterium*, and *Pseudescherichia* suggest that GDM may create a microbial environment conducive to infection (Abbott et al., 2011; Ali and Alsoub, 2021). This shift in microbiota composition can lead to a pro-inflammatory milieu, indicative of a dysbiotic state that disrupts the delicate balance necessary for neonatal immune preparedness.

FMT experiments provide crucial validation of the previously observed associations between GDM and altered microbial profiles, demonstrating a causal relationship through shifts in inflammation and gut permeability in murine models. The inflammatory response observed in mice receiving microbiota from GDM-exposed neonates, characterized by elevated proinflammatory cytokines such as IL-1β and IL-6, closely mirrors the systemic inflammatory profile observed in GDM-exposed infants (Hernández-Rodríguez et al., 2004). This finding reinforces the hypothesis that GDM-induced changes in the gut microbiota have far-reaching consequences and suggests that such microbial alterations can instigate a cascade of immune dysfunction. The notable reduction in the level of IL-10, a key anti-inflammatory cytokine, further solidifies the premise of an inflammatory state induced by the GDM-associated microbiota (Burrello et al., 2018; Sun et al., 2018). Concurrently, the downregulation of tight junction proteins (*ZO-1*, occludin, claudin-1, and mucin1) indicates compromised intestinal barrier function, which can exacerbate systemic inflammation and predispose the host to infections by allowing increased translocation of pathogens or their components, such as LPS, into the bloodstream (Ghosh et al., 2020; Paradis et al., 2021).

### 4.1 Study limitations

Several limitations of this study warrant consideration. First, the fecal samples collected represent only a snapshot of the microbial status at birth and may not fully reflect the ongoing colonization process of the gut microbiota. The dynamic nature of microbial establishment during the neonatal period suggests that longitudinal sampling would provide a more comprehensive understanding of the temporal evolution of dysbiosis associated with GDM exposure. Second, despite our efforts to control for confounding variables, we were unable to account for all potential influencing factors, including maternal dietary patterns, prenatal antibiotic use, and infant feeding modalities (breastfeeding versus formula feeding). These factors have been demonstrated to significantly impact early microbial colonization patterns and may interact with GDM-related effects in complex ways (Liu J. et al., 2024; Liu et al., 2025).

Furthermore, while we observed significant associations between the GDM-related microbiome and inflammatory markers and intestinal barrier function, we did not conduct direct pathogen challenge experiments. Consequently, our conclusion regarding “increased susceptibility to infections” is inferred from these indirect indicators rather than demonstrated through experimental infection models. The translational relevance of our murine FMT experiments, while providing mechanistic insights, may not fully recapitulate the complexity of human neonatal immune development in the context of maternal metabolic disorders. Additionally, our study primarily focused on taxonomic profiling and targeted metabolite analysis, which may not capture the full spectrum of functional alterations in the microbiome of GDM-exposed neonates. Comprehensive metagenomic, metatranscriptomic, and broader metabolomic approaches would provide deeper insights into the functional consequences of the observed dysbiosis.

### 4.2 Future research directions

Future investigations should address several key areas to build upon our findings. First, longitudinal follow-up studies extending from birth through early childhood are essential to evaluate the persistent effects of GDM on infant gut microbiota composition and infection susceptibility. Such studies would clarify whether the dysbiotic patterns observed at birth represent transient alterations or persistent ecological shifts with long-term health implications (Sarkar et al., 2021). Tracking the developmental trajectory of the microbiome alongside immunological parameters would provide valuable insights into the windows of opportunity for potential interventions (Song et al., 2023).

Second, mechanistic infection challenge experiments should be conducted to directly validate the impact of GDM-associated microbiota on host resistance to pathogenic organisms. These experiments could involve exposing gnotobiotic animals colonized with GDM or control microbiota to specific pathogens relevant to neonatal infections, such as group B *Streptococcus* or *Pseudescherichia vulneris*, to assess differences in colonization resistance, bacterial translocation, and systemic immune responses.

Third, exploration of microbiota-targeted intervention strategies represents a promising avenue for mitigating the adverse effects of GDM on offspring. Specific probiotic formulations enriched in SCFA-producing bacteria (e.g., *Bifidobacterium* and *Faecalibacterium* species), prebiotic compounds designed to selectively promote beneficial commensals, or synbiotic preparations could potentially restore microbial diversity and functional capacity in GDM-exposed neonates. Preliminary evidence suggests that such interventions may ameliorate immunological perturbations associated with early-life dysbiosis (Li et al., 2023; Beldie et al., 2024).

Finally, investigating maternal prenatal interventions for their potential protective effects on neonatal microbiome development merits consideration. These could include dietary modifications, physical activity regimens, or pharmacological approaches aimed at improving glycemic control during pregnancy. Understanding how these interventions influence maternal-fetal microbial transmission and subsequent neonatal microbial colonization patterns would provide valuable insights for developing comprehensive strategies to break the cycle of intergenerational metabolic risk transmission. Integration of multi-omics approaches, including metagenomics, metabolomics, and immunophenotyping, would enhance our understanding of the complex interplay between maternal metabolism, microbial ecology, and neonatal immune development in the context of GDM.

## 5 Conclusion

Our findings elucidate the profound impact of GDM on the neonatal gut microbiota and its subsequent implications for infection risk. The significant alterations in microbial diversity and composition observed in neonates born to GDM mothers underscore the critical role of maternal metabolic disturbances in shaping the early-life microbiome. The marked depletion of beneficial taxa, coupled with an increase in potentially pathogenic organisms, establishes a clear association between GDM-induced dysbiosis and heightened systemic inflammatory responses in affected infants. Furthermore, the reduction in SCFAs highlights the metabolic consequences of GDM, indicating potential long-term health risks for offspring. Our studies further demonstrated that these microbial and metabolic disruptions may compromise intestinal barrier function, thereby increasing susceptibility to infections. The successful translational application of FMT confirms the causal relationship between GDM-associated microbiota dysbiosis and elevated inflammatory responses, emphasizing the importance of maintaining a healthy microbial environment during pregnancy. These findings advocate for an integrative approach that prioritizes prenatal microbiota monitoring and the potential for probiotic interventions to mitigate infection risk in neonates exposed to GDM. Future research should investigate the clinical efficacy of probiotics and other microbiome-targeted therapies to promote optimal neonatal health outcomes in this vulnerable population.

## Data Availability

The raw data for 16S rRNA sequencing is stored in the NCBI Sequence Read Archive (SRA) under registration number PRJNA1242026 (https://www.ncbi.nlm.nih.gov/bioproject/PRJNA1242026). Additional data related to this study can be obtained from the corresponding author upon request.
